# Staff and Administrator Perceptions of Fall Risk Management in Home- and Community-Based Service Settings

**DOI:** 10.1093/geroni/igaa057.766

**Published:** 2020-12-16

**Authors:** Lisa Juckett, Rachael Poling

**Affiliations:** The Ohio State University, Columbus, Ohio, United States

## Abstract

Older adults who receive home- and community-based services (HCBS), such as home-delivered meals and personal care assistance, are at particular risk for falls due to their extremely high prevalence of fall risk factors. HCBS organizations and their staff are well-positioned to implement fall risk screens with HCBS clients and then refer older adults to fall prevention services as needed; however, the extent to which HCBS organizations manage fall risk has yet to be systematically examined. The purpose of this qualitative study was to explore the barriers to and facilitators of implementing fall risk screens and fall prevention service referrals in HCBS organizations. A total of 26 HCBS staff members and administrators participated in semi-structured interviews and focus groups. Qualitative data were examined using directed content analysis guided by the Consolidated Framework for Implementation Research. HCBS staff expressed that strong rapport with clients allowed for them to address fall risk proactively but were concerned that their lack of fall prevention training precluded them from effective fall risk management. HCBS administrators perceived their organization to have reliable internal communication procedures which enhanced fall prevention care coordination but believed their limited connections with fall prevention service providers served as a barrier to referring at-risk clients to appropriate care. Accordingly, HCBS stakeholders are encouraged to develop strategies, such as providing fall prevention coaching and building a network of fall prevention service providers, that account for these barriers and facilitators in future efforts to support effective fall risk management with HCBS clients.

